# Coronavirus Disease 2019 (COVID-19): Forecast of an Emerging Urgency in Pakistan

**DOI:** 10.7759/cureus.8346

**Published:** 2020-05-28

**Authors:** Rabia M Chaudhry, Asif Hanif, Muhammad Chaudhary, Sadia Minhas, Khalid Mirza, Tahira Ashraf, Syed A Gilani, Muhammad Kashif

**Affiliations:** 1 Public Health, University Institute of Public Health, University of Lahore, Lahore, PAK; 2 Oral Medicine, Akhter Saeed Medical and Dental College, Lahore, PAK; 3 Public & Environmental Health, University of Derby, Derby, GBR; 4 Oral Pathology, Akhter Saeed Medical and Dental College, Lahore, PAK; 5 Microbiology, University of Lahore, Lahore, PAK; 6 Periodontology, Akhtar Saeed Medical and Dental College, Lahore, PAK; 7 University Institute of Radiological Sciences & Medical Imaging Technology, University of Lahore, Lahore, PAK; 8 University Institute of Radiological Sciences & Medical Imaging Technology/Radiology Research Section, University of Lahore, Lahore, PAK; 9 Oral Pathology, Bakhtawar Amin Medical and Dental College, Multan, PAK

**Keywords:** covid-19, epidemiology, covid-19 forecasting, pakistan

## Abstract

Background/Aims

Novel coronavirus disease 2019 (COVID-19) is a global challenge due to little available knowledge and treatment protocols. Thus, there is a great need for collecting data related to COVID-19 from all around the world. Hence, we conducted this study, collecting daily data on COVID-19, to map the epidemiology outbreak and forecast its trajectory for May 2020.

Methodology

The data was collected from the officially released reports of the National Institute of Health (NIH), Pakistan, and the World Health Organization (WHO). The analysis was done using Statistical Package for the Social Sciences (SPSS) version 23 (IBM Corp., Armonk, NY), and forecasting was done using a simple moving average in time series modeler/expert modeler.

Results

The purpose of this study is to draw the attention of international, as well as national, governing bodies to the rapidly rising number of COVID-19 cases in Pakistan, and the urgency of evaluating the efficacy of the currently implemented strategy against COVID-19. According to this study, there is now an alarming increase in the number of COVID-19 patients in Pakistan, despite a contained spread in the beginning. The predicted number of COVID-19 cases can go over 35,000 by the end of May 2020.

Conclusion

It is crucial for governing bodies, administrators, and researchers to re-evaluate the current situation, designed policies, and implemented strategies.

## Introduction

What has now been declared as a pandemic, severe acute respiratory syndrome coronavirus 2 (SARS-CoV-2) was first reported in a livestock market in Wuhan, Hubei province of China, on December 12, 2019 [1-2]. The first case of this pandemic was presented with pneumonia of unexplained cause, and by December 29, 2019, there were 27 cases that presented with similar signs and symptoms, out of which seven were critical [3-4]. After ruling out influenza and other possible coronaviruses, it was declared that a novel zoonotic coronavirus had been the cause of this recent outbreak [3]. By January 30, 2020, the outbreak of this novel virus was so rapid and far-reaching, that the World Health Organization (WHO) announced it as a Public Health Emergency of International Concern (PHEIC) [5]. On February 12, 2020, WHO termed the disease triggered by this novel coronavirus as coronavirus disease 2019 (COVID-19) [6].

Since the outbreak of COVID-19 in December, the disease has spread to affect many countries around the globe. By April 2, 2020, the globally confirmed positive cases reached 857,641, with 42,006 deaths, and 206 countries, areas, or territories battling SARS-CoV-2 [7]. Although China was severely affected, as it was the center of the outbreak of COVID-19, the outbreak is now more concentrated in North America and Europe according to the World Health Organization COVID-19 Dashboard. However, a change is now seen in the global trend of COVID-19. After China, Europe is also starting to show a slight decline in the death rate due to COVID-19, however, South East Asia is now starting to show an increase in the growth rate of COVID-19 [7].

Pakistan is also fighting this novel coronavirus. Tackling a pandemic for which there is currently no treatment nor vaccination available and that has brought many developed countries to its knees is a great challenge for a developing country like Pakistan, which is already facing an economic crisis. Hence, the purpose of this study is to collect, summarize, and analyze the day-wise situation of COVID-19 in Pakistan, as well as the measures taken by the government of Pakistan to prevent and control it. The aim of this study is to forecast the trajectory of COVID-19 cases in Pakistan to bring the attention of governing bodies to the alarming situation of COVID-19 growth in Pakistan. Through its summarized data, this study aims to provide assistance to national and international strategists to evaluate the current strategy against COVID-19 in Pakistan and propose others if required. This study also aims to assist other researchers to compare the COVID-19 epidemiology in Pakistan with that in the rest of the world and, in the future, to conduct an After Action Review (AAR) of the measures taken and the best practices adopted against COVID-19 [8].

## Materials and methods

Data

An analysis of the daily situation of people affected by COVID-19 can help us understand the national spread of COVID-19 in Pakistan. For updates on COVID-19 cases, an open database was established nationally by NIH, Pakistan, and internationally by the WHO. Daily data were collected from NIH, Pakistan (accessed at http://covid.gov.pk/), and WHO [7-8]. The data consist of five key variables, which are laboratory tests performed, laboratory-confirmed positive cases, deaths, recoveries, and travelers screened. To gather national and provincial data, a total of seven areas was studied, including four provinces (Punjab, Sindh, Khyber Pakhtunkhwa, and Baluchistan), the Islamabad capital territory, Gilgit Baltistan, and Jammu and Azad Kashmir. The data updates were gathered daily at 12 p.m. (GMT+5) during the period of February 26, 2020, to April 3, 2020. The aim of this article is to accumulate the daily figures of COVID-19 cases in Pakistan, to get an understanding of changes in the situation of affected cases with the passage of time and forecasting the possible number of affected cases nationally and provincially.

Time series forecasting

We modeled and forecasted the cumulative trajectory of the COVID-19 epidemic in Pakistan. Forecasting means to predict the future using present and past data. The most commonly used methods for forecasting are explanatory techniques, qualitative methods, and time series algorithms. A time series is a series of discrete-time data points listed in time order [9]. Various models of time series can be used to forecast such as moving average (MA), weighted moving average (WMA), and single exponential smoothing (SES). A moving average (MA) is a commonly used technical analysis indicator in which a series of data points are averaged to analyze the data points. The MA can be a simple moving average (SMA) or an exponential moving average. An SMA is used to predict the trend by calculating the arithmetic mean of a set of values on the assumption that future observation is corresponding to past observations. The MA of the next point equals the average of the recent K observation. As K increases, most forecast relies on older data. Mathematically, SMA can be computed using the following formula [10]:

SMA = D1+D2+.....+Dk/n

Where MA, D, k, and n are moving average, observed data value, number of points period, and number of data points, respectively.

We have used SMA in a time series/expert model for our forecasting of the early trajectory of COVID-19 in a line chart in Pakistan. We have made two forecasts of COVID-19, one for April 2020 and another for May 2020, for a comparison of the change in the situation over that period of time. The forecast made for the month of April was for the early, more controlled situation in Pakistan while the May 2020 forecast is for the current, rapidly worsening situation. For the April 2020 forecast, the data were collected from February 26, 2020, to April 3, 2020, and the forecasting was made by using the SMA of “cumulative lab-tested cases positive.” While for the month of May 2020, data were collected from February 26, 2020, to April 20, 2020, and the forecasting was made using two models, that is, cumulative cases “lab tested positive cases till date” and “cumulative deaths till date.” The data analysis used Statistical Package for the Social Sciences (SPSS) version 23 (IBM Corp., Armonk, NY). For each model, forecasts started after the last non-missing in the range of the requested estimation period and at the end of the last period for which the non-missing values of all the predictors were available or at the end date of the requested forecast period, whichever was earlier.

## Results

COVID-19 change in epidemiology over time

Pakistan reported its first case on February 26, 2020. On March 10, 2020, Pueyo, an engineer, suggested a model to predict the rise in the COVID-19 cases based on either the total number of deaths or the total number of positive cases [11]. Based on this, on March 16, 2020, a journalist and an engineer, Osama Rizvi and Ahsan Zahid, respectively, suggested the trajectory of COVID-19 in Pakistan if efficient and timely preventive measures are not in place. They stated on March 16, 2020, that based on 94 true positive cases of COVID-19 in Pakistan, the likely true cases in a month could be 79,419 [12]. Fortunately, by March 29, 2020, around 15 days later, the total number of confirmed cases was 1,526, with 29 recovered and 14 deaths. Critical cases treated in the intensive care unit were 11, which contributed to 0.7% of the total cases. The daily cases receiving treatment were 1483, which amounts to 97.2% of the total cases or seven confirmed cases per million population. The fatality rate was 0.9% of the total cases and the positive rate was 1.9% of the total cases [13]. The daily situation report of COVID-19 in Pakistan, collected from the daily situation report published by the NIH, Pakistan, till April 3, 2020, is presented in Table 1 [8].

**Table 1:** Daily situation report of COVID-19 in Pakistan Note: Data collected from NIH, Pakistan. COVID-19: coronavirus disease 2019; NIH: National Institute of Health

DATE	SUSPECTED CASES		LAB RESULTS		TRAVELERS SCREENED		
	New	Total	Total Tests Performed	Total Tests Positive	New	Total	Suspects
26/2/2020	N/A*	N/A	N/A	02	N/A	N/A	N/A
29/2/2020	N/A	N/A	N/A	04	N/A	N/A	N/A
2/3/2020	N/A	N/A	N/A	05	N/A	N/A	N/A
08/3/2020	N/A	N/A	N/A	07	N/A	N/A	N/A
9/3/2020	N/A	N/A	N/A	16	N/A	N/A	N/A
10/3/2020	N/A	N/A	N/A	16	N/A	N/A	N/A
11/3/2020	14	248	422	19	21,102	873,288	26
12/3/2020	23	271	471	20	20,968	894,256	29
13/3 2020	47	318	531	21	19,286	913,542	33
14/32020	45	363	609	28	20,428	933,970	43
15/3 2020	19	382	686	31	20,676	954,646	59
16/32020	52	442	833	53	20,988	975,634	74
17/3/2020	100	533	1571	187	20,187	995,821	95
18/3/2020	127	612	1621	241	20,088	1,015,909	115
19/3/2020	126	734	1979	302	17,975	1,033,884	151
20/3/2020	478	1364	3410	461	15,398	1,049,282	170
21/3/ 2020	380	1,776	4,001	495	13911	1,063,193	186
22/3/2020	709	2574	5,225	646	14,439	1,077,632	202
23/3/2020	89	2,642	5,444	784	12,301	1,089,933	217
24/3/2020	281	2,923	5,857	887	12,301	1,102,234	232
25/3/2020	186	3,118	6,123	991	149	1,102,383	234
26/3/2020	206	3,362	6,449	1,057	N/A	1,102,383	234
27/3/2020	181	9524	7,835	1,197	N/A	1,102,383	234
28/3/2020	937	12,247	13,231	1,408	N/A	1,102,383	234
29/3/2020	1106	13,324	14,336	1,526	N/A	1,102,383	234
30/3/2020	1,121	14,445	14,748	1,625	179	1,102,562	234
31/3/2020	1,264	15,709	14,658	1,865	0	1,102,562	234
1/4/2020	1,436	17,331	15,195	2,039	0	1,102,562	234
2/4/2020	1,684	19,015	16,777	2,291	0	1,102,562	234
3/4/2020	1,798	20,813	30,308	2,450	0	1,102,562	234

**Figure 1:**
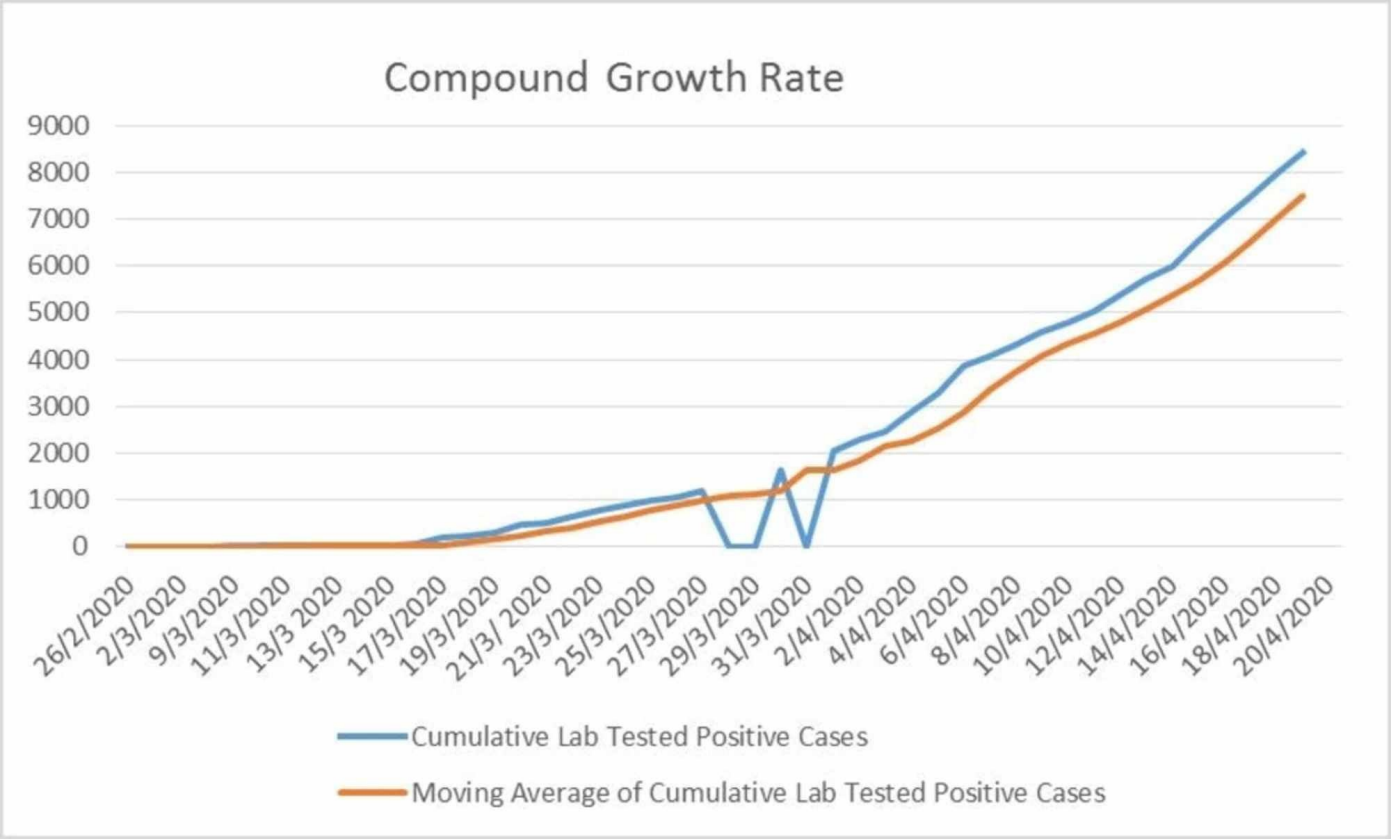
Graph of the compound growth rate of COVID-19 positive cases in Pakistan COVID-19: coronavirus disease 2019

However, on April 3, 2020, the prime minister of Pakistan announced the reopening of the China-Pakistan Economic Corridor (CPEC) project and construction industry from April 4, 2020, considering the rising economic pressures [14]. There has been a slow but controlled increase through the month of March 2020 in Pakistan, but a rapid increase has been observed since the beginning of April 2020. The change in the growth rate of COVID-19 ever since its outbreak is mentioned in Figure 1. The graph of the compound growth rate of COVID-19 is generated by calculating the cumulative COVID-19 positive cases as of April 20, 2020, in Pakistan. The data were collected from the official statistics shared by the NIH, Pakistan.

The rapid and upwards trend in the COVID-19 growth rate change nationally can be observed more in detail at the regional level. The areas that are at the center of economic activities, as well as more populous areas, can be seen as more affected by the disease. Table 2 shows the regional statistics of COVID-19 in Pakistan up till April 16, 2020, collected from the NIH, Pakistan [8].

**Table 2:** Regional statistics of COVID-19 in Pakistan COVID-19: coronavirus disease 2019

	Cumulative Suspected Cases	Cumulative Tests Performed	Cumulative Tests Positive Cases	Cumulative Tests Positive %	Cumulative Cases Recovered	Cumulative Cases Expired
Pakistan	66,691	84,704	7025	8.3	1765	135
Punjab	29,326	48,381	3276	6.8	630	36
Sindh	18,900	18,900	2008	10.6	576	45
Khyber Pakhtunkhwa	5,758	4,885	993	20.3	205	45
Gilgit Baltistan	4,490	4,427	303	6.8	142	5
Baluchistan	1,932	1,826	245	13.4	183	3
Islamabad Capital Territory	5,150	5,150	154	3.0	20	1
Azad Jammu Kashmir	1,135	1,135	46	4.1	9	0

Forecasting

**Figure 2:**
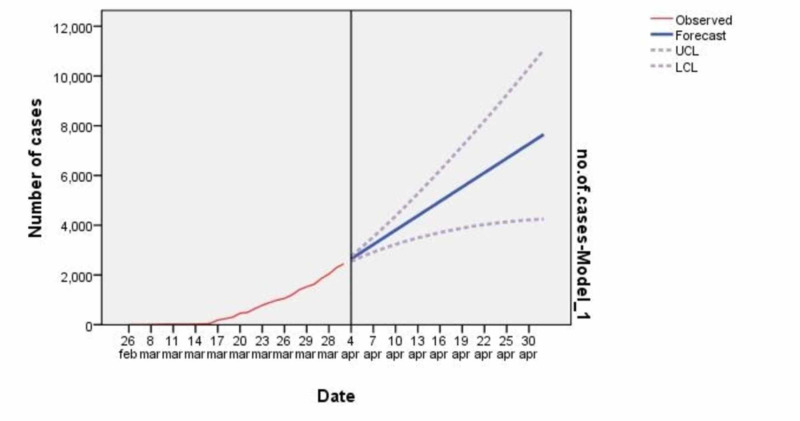
Forecasting of COVID-19 from April 4 till April 30 NOTE: For each model, forecasts start after the last non-missing in the range of the requested estimation period and ends at the last period for which the non-missing values of all the predictors are available or at the end date of the requested forecast period, whichever is earlier.

In this study, we have made two forecasts, which are discussed ahead. First, for the month of April 2020, which was based on the cumulative lab-tested positive cases of COVID-19 in Pakistan from February 26, 2020, to April 3, 2020. However, according to our analysis of data using SMA in the time series model, we predicted that there could be around 8,000 cases of COVID-19 in the country till April 30, 2020, which is in contrast to the prediction of Osama Rizvi and Ahsan Zahid. As of April 19, 2020, the total number of lab-tested positive cases of COVID-19 in Pakistan is 8,420. The line chart of the forecast we made for the month of April 2020 of COVID-19 cases in Pakistan is given in Figure 2.

The second forecast we have made using the SMA of the time series model in SPSS version 23 is for the month of May 2020. There are two models that are used for this forecasting, the total number of lab-tested positive cases and the total number of confirmed deaths. This forecast is made by collecting data from February 26, 2020, to April 20, 2020, and hence accounts for the change in the rapid increase in COVID-19 cases in the month of April. Control limits were calculated by:

Estimating standard deviation, σ, of the collected data,

Multiplication of that number by three,

Addition of (3 x σ to the average) for the upper control limit (UCL) and subtraction of (3 x σ from the average) for the lower control limit (LCL).

Mathematically, the formula of the control limits is:

CL = average ±3*σ

Hence, according to our calculation, the forecast for the month of May 2020 of COVID-19 cases in Pakistan, using two models, is mentioned in Table 3.

**Table 3:** Forecasting of COVID-19 with UCL and LCL from April 21, 2020, till May 31, 2020, using two different models, “Lab Tested True Positive Cases” and “Total Number of Deaths” to forecast the trajectory of COVID-19 cases in Pakistan UCL: upper control limit; LCL: lower control limit; COVID-19: coronavirus disease 2019

	Model					
	Lab-tested positive cases-Model_1			No of deaths-Model_2		
	Forecast	UCL	LCL	Forecast	UCL	LCL
21-Apr-20	9818	10026	9610	206	210	201
22-Apr-20	10448	10810	10086	222	230	214
23-Apr-20	11079	11619	10538	240	250	230
24-Apr-20	11709	12451	10967	257	269	244
25-Apr-20	12339	13301	11377	273	289	256
26-Apr-20	12970	14170	11769	291	311	270
27-Apr-20	13600	15056	12144	310	334	285
28-Apr-20	14231	15958	12503	328	356	299
29-Apr-20	14861	16875	12847	346	379	313
30-Apr-20	15491	17806	13177	365	403	327
01-May-20	16122	18750	13494	385	427	342
02-May-20	16752	19707	13797	404	452	357
03-May-20	17383	20677	14088	424	477	371
04-May-20	18013	21660	14366	445	503	386
05-May-20	18643	22653	14633	466	530	402
06-May-20	19274	23658	14889	487	557	417
07-May-20	19904	24674	15134	508	584	432
08-May-20	20534	25701	15368	530	612	448
09-May-20	21165	26738	15592	553	641	464
10-May-20	21795	27785	15805	575	670	480
11-May-20	22426	28843	16009	598	699	497
12-May-20	23056	29909	16203	621	729	513
13-May-20	23686	30986	16387	645	760	530
14-May-20	24317	32071	16562	669	791	547
15-May-20	24947	33166	16729	693	822	564
16-May-20	25578	34269	16886	718	854	581
17-May-20	26208	35382	17034	743	887	599
18-May-20	26838	36502	17174	768	920	617
19-May-20	27469	37632	17306	794	953	635
20-May-20	28099	38769	17429	820	987	653
21-May-20	28730	39915	17544	847	1022	672
22-May-20	29360	41069	17651	874	1057	690
23-May-20	29990	42231	17750	901	1092	709
24-May-20	30621	43400	17841	928	1128	729
25-May-20	31251	44577	17925	956	1164	748
26-May-20	31881	45762	18001	984	1201	768
27-May-20	32512	46954	18069	1013	1238	788
28-May-20	33142	48154	18131	1042	1276	808
29-May-20	33773	49361	18184	1071	1314	828
30-May-20	34403	50575	18231	1101	1353	849
31-May-20	35033	51796	18271	1131	1392	870
	Model					
	Lab-tested positive cases-Model_1			No of deaths-Model_2		
	Forecast	UCL	LCL	Forecast	UCL	LCL
21-Apr-20	9818	10026	9610	206	210	201
22-Apr-20	10448	10810	10086	222	230	214
23-Apr-20	11079	11619	10538	240	250	230
24-Apr-20	11709	12451	10967	257	269	244
25-Apr-20	12339	13301	11377	273	289	256
26-Apr-20	12970	14170	11769	291	311	270
27-Apr-20	13600	15056	12144	310	334	285
28-Apr-20	14231	15958	12503	328	356	299
29-Apr-20	14861	16875	12847	346	379	313
30-Apr-20	15491	17806	13177	365	403	327
01-May-20	16122	18750	13494	385	427	342
02-May-20	16752	19707	13797	404	452	357
03-May-20	17383	20677	14088	424	477	371
04-May-20	18013	21660	14366	445	503	386
05-May-20	18643	22653	14633	466	530	402
06-May-20	19274	23658	14889	487	557	417
07-May-20	19904	24674	15134	508	584	432
08-May-20	20534	25701	15368	530	612	448
09-May-20	21165	26738	15592	553	641	464
10-May-20	21795	27785	15805	575	670	480
11-May-20	22426	28843	16009	598	699	497
12-May-20	23056	29909	16203	621	729	513
13-May-20	23686	30986	16387	645	760	530
14-May-20	24317	32071	16562	669	791	547
15-May-20	24947	33166	16729	693	822	564
16-May-20	25578	34269	16886	718	854	581
17-May-20	26208	35382	17034	743	887	599
18-May-20	26838	36502	17174	768	920	617
19-May-20	27469	37632	17306	794	953	635
20-May-20	28099	38769	17429	820	987	653
21-May-20	28730	39915	17544	847	1022	672
22-May-20	29360	41069	17651	874	1057	690
23-May-20	29990	42231	17750	901	1092	709
24-May-20	30621	43400	17841	928	1128	729
25-May-20	31251	44577	17925	956	1164	748
26-May-20	31881	45762	18001	984	1201	768
27-May-20	32512	46954	18069	1013	1238	788
28-May-20	33142	48154	18131	1042	1276	808
29-May-20	33773	49361	18184	1071	1314	828
30-May-20	34403	50575	18231	1101	1353	849
31-May-20	35033	51796	18271	1131	1392	870

**Figure 3:**
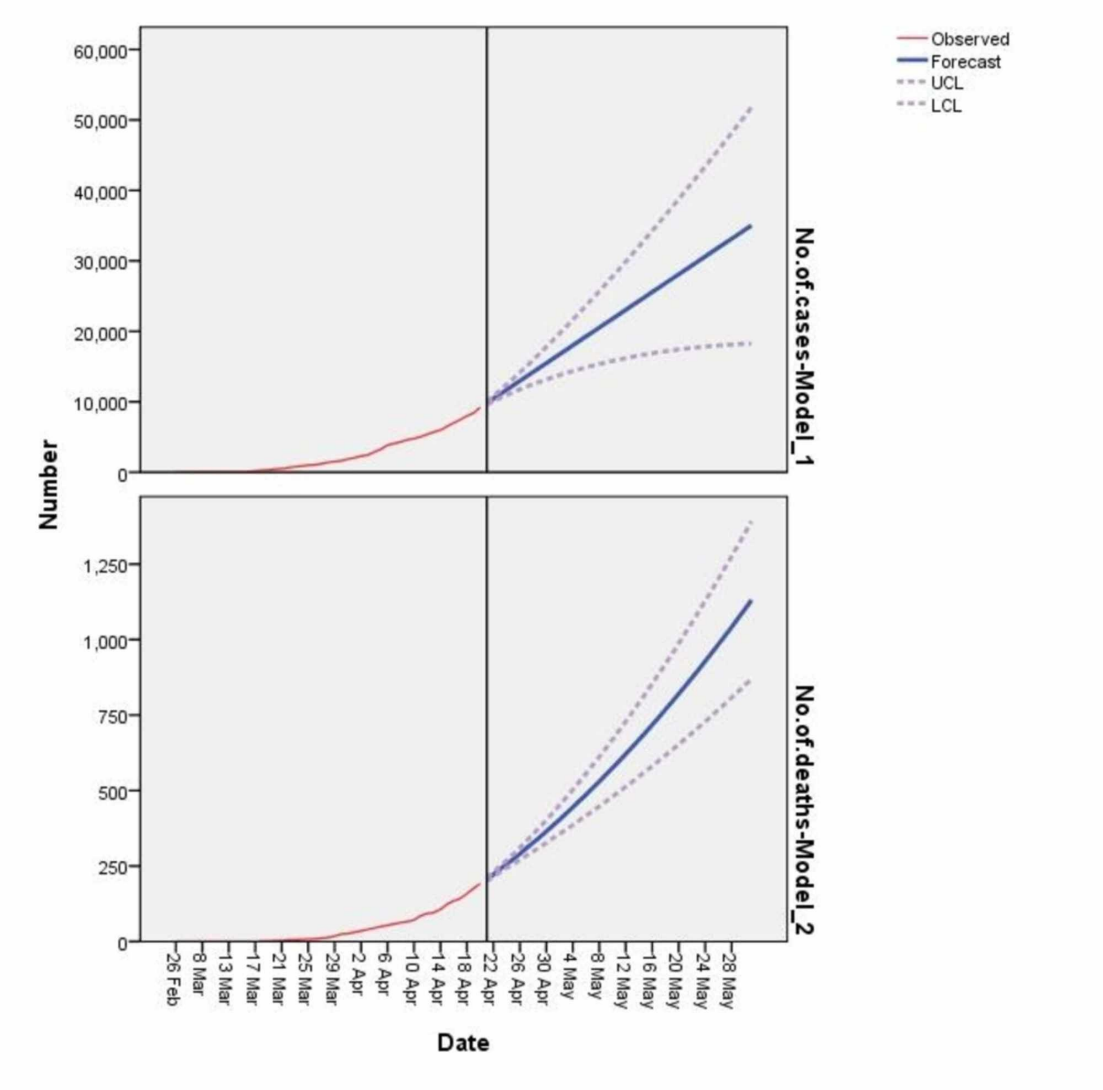
Forecasting of COVID-19 from April 22, 2020, to May 31, 2020 According to the figure, there may be around 35,000 cases of COVID-19 in Pakistan by the end of May 2020. COVID-19: coronavirus disease 2019

As we have used two models, the total number of lab-tested true positive cases and the total number of deaths, we predict that the total number of lab-tested true positive cases can multiply up to almost five times and go as high as around 35,000 cases in the month of May 2020. While based on 192 cumulative deaths as of April 20, 2020, our forecast for the number of deaths is around 1100 in the next month, which is also around seven times higher than the current death rate (Figure 3).

## Discussion

An ongoing study by Roser et al. on Coronavirus Disease (COVID-19) - Statistics and Research is currently sharing and comparing updated global data from the database of the European Center for Disease Control (CDC) [13]. Following is the comparison of COVID-19 situation among China, the USA, and Pakistan during the time period of December 31, 2019, to March 30, 2020. China reported its first COVID-19 case in December 2020 while the USA had it's first confirmed case on January 20, 2020, and Pakistan had its first case almost a month after the USA on February 26, 2020, despite sharing a border, CPEC, and extensive trade with China. In April 2020, China, after experiencing its peak in the month of February 2020, is now on the decline, while the USA, on the other hand, is currently headed towards its peak, almost two months after its first reported case. Currently, the USA is the most affected country in the world while Pakistan shows the least spread of COVID-19 among the three countries [7-8].

However, will the COVID-19 growth rate reduce or stay the same in Pakistan in the future as well? Is the situation still as safe as it has been? These are the two questions we intend to bring to attention in this article, considering our forecast of COVID-19 cases in Pakistan. As for the first question, within a week, the per-day cases of Pakistan has jumped from 269 to 533 on April 21, 2020, while the total number of lab-tested positive cases has gone from 5,985 to 9,749. This pattern is similar to the trend in China on January 26, 2020, in the USA on March 11, 2020, and in Italy on March 5, 2020, where, after the cases once reached around 500 per day, the incline in the exponential growth followed right after, with reported cases in the thousands per day [8,13]. According to a study being conducted by Handley, when the compound growth rate hits 35%, it takes 2.5 days for the COVID-19 cases to double and 3.5 days when it reaches 22% [14]. As of April 21, 2020, Pakistan has a compound growth rate of 17.3% of COVID-19, which means that if not controlled, the cases in Pakistan will soon be doubling in three to four days [14-15]. COVID-19 growth as of April 23, 2020, is already rather faster and higher than we originally predicted in Table 3 based on the data collected from February 26, 2020, to April 3, 2020. With the recent rapid increase in the growth of COVID-19, the cases by the end of April 2020 can go much higher than our initial prediction of 8,000, which is already a great threat and challenge for the governing bodies in Pakistan. But, if the situation continues, according to our forecast, the COVID-19 number can actually multiply up to seven times. Based on our two models of the forecast, using SMA in the time series model, we predict that the total lab-tested positive cases by the end of May can go over 35,000, and deaths can be over 1100 based on the total number of confirmed deaths. Hence, according to our study, it may not be wrong to say that the country seems to be moving towards facing an alarming high in COVID-19 trends in the future.

For the second question, on April 14, 2020, the Prime Minister of Pakistan has also announced to open the skilled labor industry along with the CPEC and construction industry, which will further loosen the lockdown and the protective effect of social distancing against COVID-19. Succumbing to economic pressure, the traders association in Karachi has also announced to reopen businesses in three out of four provinces, Sindh, southwestern Baluchistan, and northwestern Khyber Pakhtunkhwa (KP), on the same day [16]. Later in the day, religious relics also announced to open the places of worship for five daily congregational prayers, as well as extra congregational prayers, during the holy month of Ramadan while observing preventive protocols [17]. How can these decisions affect COVID-19 in Pakistan? Forecasting means to predict the future based on data of the past and present. Generally, forecasting is done using qualitative methods, explanatory techniques, and time series algorithms. Osama Rizvi and Ahsan Zahid, on March 16, 2020, using the Pueyo model of forecasting COVID-19, suggested that based on 94 true positive cases of COVID-19 in Pakistan, the trajectory of the disease could be up to 79,419 in a month [12]. However, Thomas Pueyo has also suggested a method for the forecasting of COVID-19 specifically [11]. A study by Elmousalami and Hassanien discusses the forecasting of global COVID-19 cases based on the algorithmic trend of COVID-19 cases in the world using the mathematical formula mentioned below [18]:

N_d_ = (1+ E*P)^d^ N_0_

Where,

N_d_: the expected number of confirmed cases in the future

E: average number of people someone infected is exposed to each day

P: the probability of each exposure becoming an infection

N_0_: the initial number of cases at a given time

d: the number of days between the given time and the future time

According to this formula, Nd (number of positive cases) is directly proportional to E and P, which means that Nd is inversely proportional to social distancing and preventive measures like lockdown [19]. This means that with industries opening, fewer restrictions, and more social gatherings due to religious reasons, it may further accelerate the already rapidly increasing growth rate of COVID-19 in the country. This assumption is based on this study that shows that countries that did not implement social distancing showed a compound growth rate of 25%, a finding consistent in Italy, France, Spain, and the UK. On the contrary, all the countries successful to contain the spread shared the same striking feature of restricted person-to-person exposure [18]. Hence, with the current state of affairs of the country, we would like to warn of a great danger of exponential growth in the COVID-19 cases in Pakistan. We forecast over 35,000 COVID-19 cases by the end of May 2020, as well as a health and economic crisis for a developing country.

A WHO representative on March 14, 2020, declared the response of Pakistan as one of the best national responses [19]. This may be supported by the fact that the COVID-19 growth rate has been slower than expected in the country. However, the total foreign transmission of COVID-19 in Pakistan is estimated to be about 46.6%, which goes to show that Pakistan’s border prevention strategy could have been improved and still needs to be revisited for future courses. On the other hand, local transmission of COVID-19 accounts for 53.4% after a month of lockdown in the country. The question of whether the reason for this relatively slower spread is only due to timely, efficient measures, or other factors are involved has yet to be answered. The role of other factors like the increased ratio of the younger population, better immune status, warm climate, or under testing has been associated with the slow spread of COVID-19. Pakistan is a country of warm climate, and under-reporting of COVID-19 is being brought to attention; moreover, contrary to many other countries COVID-19 in Pakistan is being reported more in the younger age group [20-22]. However, despite the slower and more contained numbers, there is currently a surge in COVID-19 cases. Pakistan, as a developing country under debt, with a relatively underdeveloped health care infrastructure and lack of financial resources, may not seem to be able to give an emergency response like that of China, especially when other developed countries seem to be struggling to battle this pandemic too. So, in our opinion, for researchers, administrators, and policymakers, it is worth monitoring the national response of the country when the outbreak worsens, considering its earlier efficient response for future studies and analysis.

Hence, considering the exponential growth of COVID-19 cases, along with recent economic decisions made to loosen the lockdown, Pakistan seems to be on the verge of facing a COVID-19 outbreak challenge. So, through this study, we aim to forecast a possible outbreak in the country. The purpose of this forecast, as well as a study of the current epidemiology of COVID-19 and the preventive measures taken by Pakistan, is to draw the attention of both national as well international governing bodies, administrators, and researchers to the re-evaluation and re-orientation of the current implemented COVID-19 prevention strategies, as well as the preparedness of the country for a potential national health crisis. Through this study, we also invite researchers to further investigate the effect of these factors on the epidemiological spread of COVID-19.

## Conclusions

The government of Pakistan started taking preventive measures against COVID-19 before its outbreak in the country. However, there has now been a rapid increase in the number of confirmed positive cases of COVID-19 in Pakistan. The growth seems to be higher than what our study predicted for the month of April, that is, around 8,000 cases but slower than predicted by earlier researchers. According to our forecast, COVID-19 cases may surge over 35,000 in May 2020. Hence, the upcoming month is crucial in the epidemiological spread of COVID-19, as well as the preventive decisions and policies Pakistan makes. How will Pakistan manage both its COVID-19 cases and its economy is a situation to monitor. Also, a question that should be further investigated is the cause of the less than the expected spread of COVID-19 in Pakistan, as compared to other active outbreak countries.
